# Cardiac Dimensions in Normal Pregnancy: A Prospective Study

**DOI:** 10.7759/cureus.40868

**Published:** 2023-06-23

**Authors:** Ugoeze N Iloeje, Daniel Jesurobo, Abaram C Mankwe, Anthony G Kweki, Henry O Aiwuyo, Oluwaseye M Oladimeji, Isioma Emenena, Maclean R Akpa, O J Odia

**Affiliations:** 1 Internal Medicine/Cardiology, Federal Medical Centre, Yenagoa, NGA; 2 Internal Medicine, Federal Medical Centre, Yenagoa, NGA; 3 Internal Medicine/Cardiology, Colchester Hospital, East Suffolk and North Essex NHS Foundation Trust (ESNEFT), Colchester, GBR; 4 Internal Medicine, Brookdale University Hospital Medical Center, Brooklyn, USA; 5 Medicine, Lagos State University Teaching Hospital, Lagos, NGA; 6 Internal Medicine/Gastroenterology, Delta State University Teaching Hospital, Oghara, NGA; 7 Internal Medicine, University of Port Harcourt Teaching Hospital, Port Harcourt, NGA

**Keywords:** cardiovascular disorders, echocardiography, prospective study, pregnancy, cardiac dimensions

## Abstract

Background: Pregnancy, a unique physiologic state, is associated with several changes in the various body systems. The cardiovascular system is one of the systems affected, with chronic volume overload being one of the characteristic changes experienced during pregnancy. Cardiovascular disease in pregnancy is the leading cause of non-obstetric maternal death worldwide.

Aim: This study aims to determine and describe the changes in left and right ventricular and atrial sizes in systole and diastole in the course of normal pregnancy.

Methods and materials: A cohort study was conducted among healthy pregnant women between the age of 18 and 40 who attended the antenatal clinic of Federal Medical Centre (FMC), Yenagoa, Bayelsa State. Fifty women were recruited during the first trimester (T1) of pregnancy and followed up until six weeks postpartum. Ethical approval was obtained from the Research Ethics Committee of Federal Medical Centre, Yenagoa, with approval number FMCY/REC/ECC/2019/JAN/150. Clinical evaluation, hematologic, biochemical, and anthropometric assessments, and two-dimensional M-mode and Doppler echocardiography were done for the participants in each trimester of pregnancy and at six weeks postpartum. The clinical and echocardiographic parameters were analyzed using Statistical Package for the Social Sciences (SPSS) version 22.0 (IBM Corp., Armonk, NY, USA).

Results: The mean trend of left ventricular posterior wall thickness in diastole (LVPWd) and left ventricular posterior wall thickness in systole (LVPWs) increased progressively from the first to third trimester (T3) (not statistically significant) but dropped toward initial values in postpartum to the level that was statistically significant for LVWPd alone when compared to baseline first trimester values. The left atrial diameter in systole (LADs) was largest in the third trimester, and the left atrial volume index (LAVI) and right ventricular basal diameter (RVD1) also showed a similar trend. The left ventricular internal diameter (LVID) in both systole and diastole increased progressively from the first to the third trimesters, but the increase was only statistically significant between the third trimester (T3) and the first trimester (T1). The right atrial diameter (RAD) and right atrial volume (RAV) also increased progressively from the first to the third trimesters, but the increase was only statistically significant between the third trimester (T3) and the first trimester (T1).

Conclusion: Changes were noticed in the cardiac chamber sizes during pregnancy. However, this reversed back to levels similar to the first trimester during the postpartum period. To aid in the early detection and treatment of cardiovascular disorders in pregnancy, screening of apparently healthy pregnant women who later developed complaints is advised as cardiovascular changes could be significant during pregnancy.

## Introduction

Pregnancy is a normal physiologic state that independently poses a challenge to the cardiovascular system with resultant usually reversible changes in a woman's cardiovascular system with structural remodeling and improved functional performance [1-3]. Due to the dramatic hemodynamic changes that occur in pregnancy, cardiac complications can also occur in the postpartum period, which is defined as six weeks following delivery [4]. Cardiovascular disease may also present for the first time during pregnancy [5].

A pregnancy that is uncomplicated with any chronic condition known to have cardiovascular effects such as diabetes, human immunodeficiency virus (HIV), sickle cell disease, asthma, or systemic lupus erythematosus (SLE) is referred to as a normal pregnancy [6,7]. Increased cardiac output, stroke volume, increased left ventricular wall thickness, and a decline in blood pressure and systemic vascular resistance are some maternal cardiovascular adaptations that accompany a normal pregnancy [3,7,8]. It is important to note that cardiac changes that occur during pregnancy can mimic cardiovascular disorders [9]. The changes are mechanisms by which the body copes with the increased metabolic demands of the mother and fetus to ensure adequate uteroplacental circulation for fetal growth and development.

Maternal mortality has decreased by 34% worldwide over the last decade. However, in developing countries like Nigeria, the maternal mortality rate is still increasing (576 per 100,000 live births as of 2022), with cardiovascular disorders complicating pregnancies contributing to these poor outcomes. Nigeria ranks the fourth highest in maternal mortality rate in the world [10,11]. Maternal cardiovascular disease (present in 2% of all pregnancies) is the leading cause of non-obstetric death during pregnancy worldwide. A Nigeria study however shows findings as high as 2.9 per 1,000 [1,12,13].

An increase in cardiac output is the prominent physiologic change seen in normal pregnancy. The increase can be attributed to a rise in three factors: an increase in red cell mass, an increase in heart rate, and a hormone-mediated increase in plasma volume [14,15]. Adeyeye et al. [1] in a study conducted in southwestern Nigeria reported a steady statistically significant increase in cardiac output from the early second trimester to the mid-third trimester with a mean of 5.61 ± 0.8 L/minute to 7.05 ± 0.94 L/minute compared to their control of 4.52 ± 0.8 L/minute (p < 0.05).

In a study by Clapp et al. [16], 15 nulliparous and 15 parous women were investigated before pregnancy, throughout gestation, and postpartum (up to 52 weeks postpartum). They found increases in cardiac output of 2.2 ± 0.2 L/minute, which peaked at 24 weeks, and these values were significantly greater in pregnant women compared to lower rates in the pre-pregnant state. Postpartum, the values gradually returned toward baseline values but remained significantly different from pre-pregnancy values in both groups at one year [16]. A report by Kumar et al. [17] stated that cardiac output increases with gestational age and that this increase is driven by increased heart rate in normal pregnancy.

Tso et al. [7], in a retrospective analysis of normal echocardiograms of 121 healthy women (97 pregnant and 24 non-pregnant), showed that all four cardiac chambers enlarged significantly in pregnant women compared to the non-pregnant ones, with the left atrium being the first chamber to enlarge.

This study seeks to explore the functional and structural cardiac dimensional changes associated with a healthy pregnancy to describe the possible mechanisms involved.

## Materials and methods

A cohort observational longitudinal study was conducted at Federal Medical Centre (FMC), Yenagoa. The study was conducted at the antenatal clinic and cardiac care unit of Federal Medical Centre (FMC), Yenagoa, Bayelsa State, Nigeria. The antenatal care clinic of the department of obstetrics and gynecology caters to about 48 pregnant women weekly, while the cardiac care unit caters to both inpatients and outpatients. It is the site of cardiac investigations such as electrocardiogram (ECG) and echocardiography and serves about 40 patients weekly.

Using the formula of Kish [18], an estimated sample size of 50 was obtained. A simple random sampling using a ballot was used to recruit pregnant women between the ages of 18 and 40 years attending the antenatal clinic of FMC Yenagoa. The definition of a normal pregnant woman included those without a history of hypertension, preeclampsia, diabetes or gestational diabetes mellitus (GDM), systemic lupus erythematosus (SLE), human immunodeficiency virus (HIV), sickle cell disease or asthma, cardiac disease, or any known systemic disease and not on treatment for any of these [6,7]. Fifty pregnant women were recruited in the first trimester (T1) and reviewed with a similar echo protocol in the second trimester and third trimester (T3). The women were studied with a similar echo protocol six weeks after delivery at the postnatal clinic.

Ethical approval for the study was obtained from the Research Ethics Committee of Federal Medical Centre, Yenagoa, with approval number FMCY/REC/ECC/2019/JAN/150.

A structured interviewer-administered questionnaire was used to obtain relevant demographic, social, and medical data from the participants of this study. Thorough physical examination, anthropometric measurements, and full cardiovascular examination were done for all study subjects.

A transthoracic M-mode, two-dimensional, and Doppler echocardiography was performed on all subjects recruited into the study [19]. This was done using Siemens Acuson Cypress (Siemens, Munich, Germany) plus a diagnostic ultrasound system. Calculations were made using the internal analysis software of the echo machine. Two-dimensional views were used to access real-time morphological features and also as a reference for the selection of the M-mode beam.

Echocardiographic measurement was according to the American Society of Echocardiography recommendations. Left ventricular internal diameter (LVID), interventricular septal thickness, and left ventricular posterior wall thickness in diastole (LVPWd) and left ventricular posterior wall thickness in systole (LVPWs) were measured [19]. The subjects were examined in the left lateral decubitus position using parasternal and apical views. Echocardiography was done by the corresponding author and cross-checked by research assistants.

Data entry and analysis were done using Statistical Package for the Social Sciences (SPSS) version 22.0 (IBM Corp., Armonk, NY, USA). Categorical variables were summarized and presented using frequencies and proportions. Continuous variables were summarized using mean ± standard deviation (SD) after a test of normality (Shapiro-Wilk test) was done and they were found to be normally distributed. Cardiac sizes in systole and diastole measured across the three trimesters of pregnancy and postpartum were also found to be normally distributed and summarized using the mean and standard deviation. Changes in cardiac sizes were explored using the paired t-test. The changes (differences) were estimated concerning values obtained in the first trimester of pregnancy (baseline). The mean difference between the first and second trimester readings and third trimester readings, and first trimester and postpartum readings were calculated with their 95% confidence interval (CI). A significant difference was set at p-value < 0.05.

## Results

Sociodemographic characteristics of pregnant women in the study

Of the 50 women recruited for the study, 49 completed the study, giving a completion rate of 98%. A study participant relocated out of the state during the study, resulting in a single dropout recorded. The sociodemographic characteristics of pregnant women in the study are represented in Table 1.

**Table 1:** Sociodemographic characteristics of the study participants SD: standard deviation

Characteristics	Frequency (N = 49)	Percentage (%)
Age group (years)		
<25	5	10.2
25-29	12	24.5
30-34	21	42.9
>35	11	22.4
Mean age ± SD in years	31.4 ± 4.9	
Marital status		
Single	4	8.2
Married	45	91.8
Educational level		
Secondary	6	12.2
Tertiary	43	87.8
Occupation		
Housewife	15	30.6
Business	5	10.2
Public servant	29	59.2
Religion		
Christian	48	98
Muslim	1	2

Past medical, family, and obstetrics history

Twenty-six (53.1%), 17 (34.7%), and six (12.2%) women were multiparous, nulliparous, and primiparous, respectively (Table 2). No history of chronic illness was reported among participants; however, a family history of chronic illnesses was reported among 27 (55.1%) women. A family history of hypertension was reported in 22 (44.9%) women, and a family history of diabetes mellitus was reported in 13 (26.5%) women (Table 2).

**Table 2:** Past medical, family, and obstetric history related to cardiovascular health among the study participants *More than one option is applicable.

Characteristics	Frequency (N = 49)	Percentage (%)
Parity		
Nulliparous	17	34.7
Primiparous	6	12.2
Multiparous	26	53.1
Family history of chronic illness		
Yes	27	55.1
No	22	44.9
Family history of hypertension	22	44.9
Family history of diabetes mellitus	13	26.5
Family member with hypertension*	(N = 22)	
Father	15	68.2
Mother	10	45.5
Family history of diabetes*	(N = 13)	
Father	5	38.5
Mother	9	69.2

Dimensions of the left and right ventricles and atria in systole and diastole in normal pregnancy among the study participants

**Figure 1:**
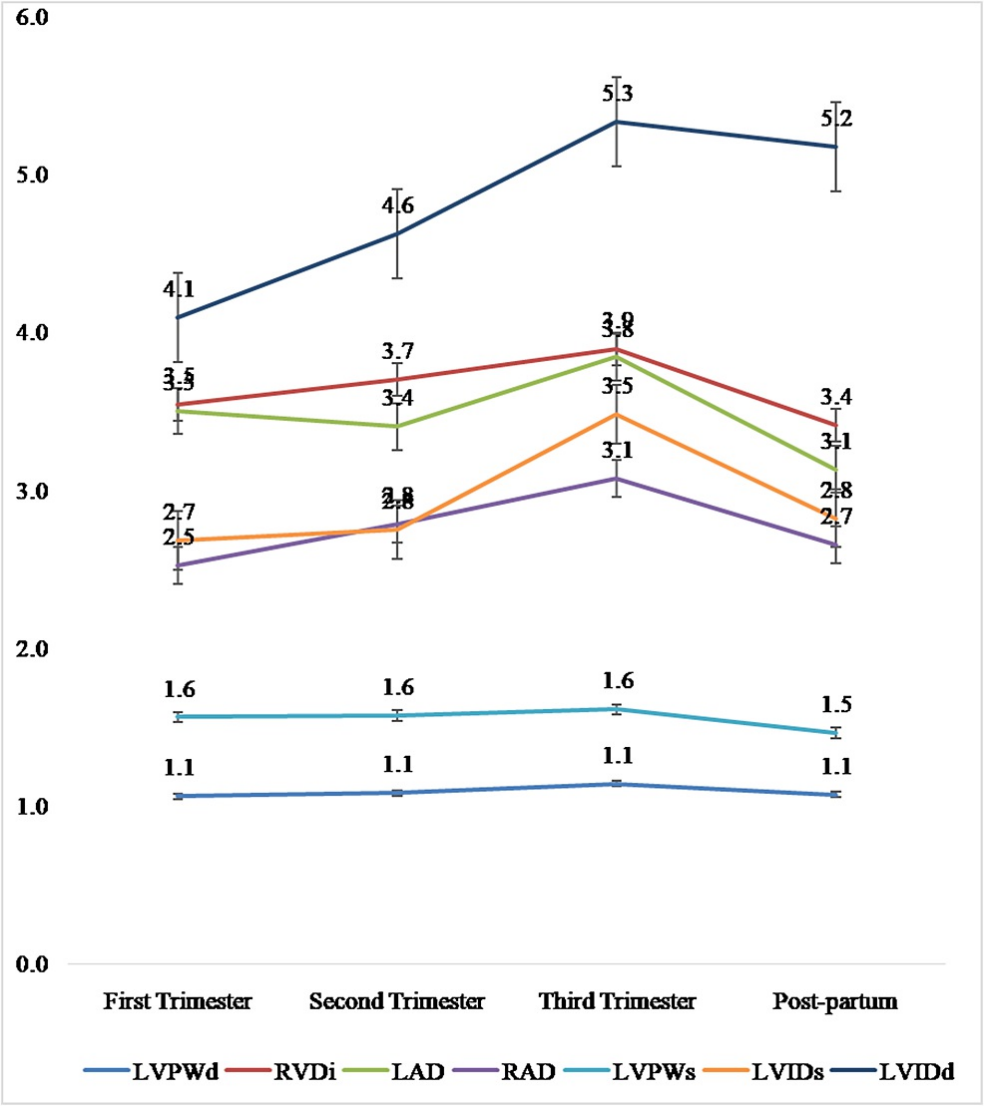
Line graph showing the mean values of cardiac sizes (LVPWd, RVD1, LAD, RAD, LVPWs, LVIDs, and LVIDd) in the first, second, and third trimesters and postpartum LVPWd: left ventricular posterior wall in diastole, RVD1: right ventricular basal diameter (basal diameter), LAD: left atrial diameter, RAD: right atrial diameter, LVPWs: left ventricular posterior wall in systole, LVIDs: left ventricular internal diameter in systole, LVIDd: left ventricular internal diameter in diastole

**Figure 2:**
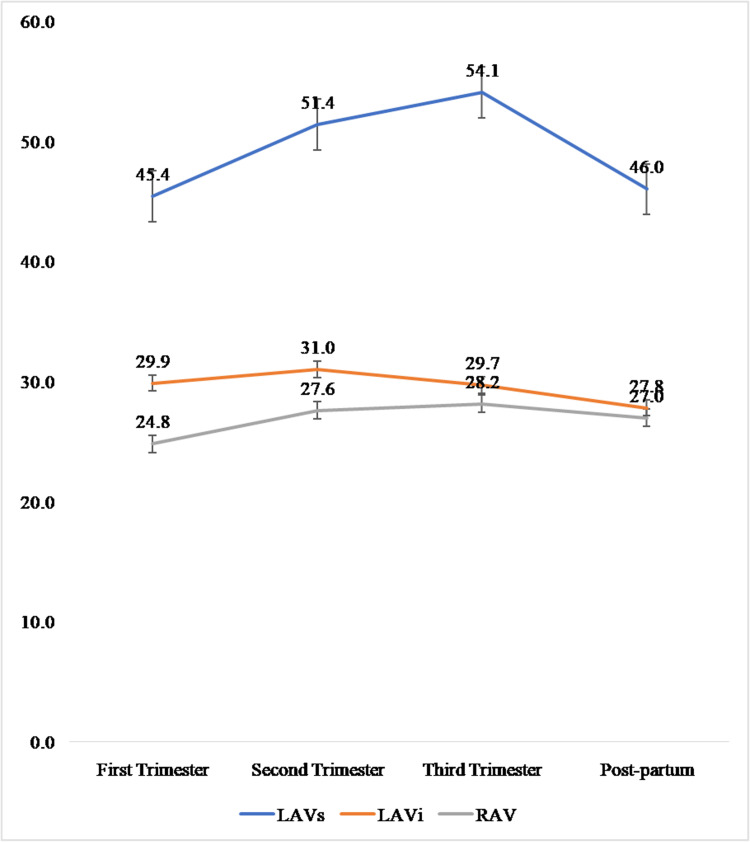
Line graph showing the mean values of cardiac sizes (LAVs, LAVI, and RAV) in the first, second, and third trimesters and postpartum in normal pregnancy LAVs: left atrial volume in systole, LAVI: left atrial volume index, RAV: right atrial volume

The mean dimensions of left ventricular posterior wall thickness in systole (LVPWs) in the first, second, and third trimesters were 1.56 ± 0.28 cm, 1.57 ± 0.33 cm, and 1.61 ± 0.37 cm, respectively (Table 3). The mean LVPWs fell to 1.46 ± 0.18 cm in the postpartum period (Table 3, Figure 1). The mean values of left ventricular posterior wall thickness in diastole (LVPWd) were similar at 1.06 ± 0.22 cm, 1.08 ± 0.23 cm, 1.14 ± 0.21 cm, and 1.07 ± 0.15 cm in the first, second, and third trimesters and postpartum period, respectively (Table 3, Figure 1). The mean dimensions for left atrial volume in systole (LAVs) and right atrial volume (RAV) peaked in the third trimester of pregnancy at 54.09 ± 5.3 mL and 28.17 ± 5.41 mL, respectively (Table 3, Figure 2). All cardiac dimensions measured increased slightly from the first to the second, to the third trimester. The mean dimensions of RAV followed the trend of other cardiac sizes, increasing gradually from 24.82 ± 4.77 mL to 27.60 ± 5.41 mL, to 28.17 ± 5.41 mL in the first, second, and third trimesters, respectively. It later fell to a value slightly higher (27.00 ± 4.67 mL) than its first trimester value in the postpartum period (Table 3, Figure 2).

**Table 3:** Cardiac dimensions in systole and diastole in normal pregnancy in the study participants LVPWd (cm): left ventricular posterior wall in diastole, LVPWs: left ventricular posterior wall in systole, LAD (cm): left atrial diameter, LVIDs: left ventricular internal diameter in systole, LVIDd: left ventricular internal diameter in diastole, LAVs: left atrial volume in systole, LAVI: left atrial volume index, RVD1 (cm): right ventricular basal diameter (basal diameter), RAD (cm): right atrial diameter, RAV (cm): right atrial volume, SD; standard deviation

Cardiac parameters	Cardiac sizes (mean ± SD)				p-value
	First trimester	Second trimester	Third trimester	Postpartum	
LVPWd (cm)	1.06 ± 0.22	1.08 ± 0.23	1.14 ± 0.21	1.07 ± 0.15	0.214
LVPWs (cm)	1.56 ± 0.28	1.57 ± 0.33	1.61 ± 0.37	1.46 ± 0.18	0.085
LAD (cm)	3.51 ± 0.2	3.74 ± 0.33	3.84 ± 0.32	3.13 ± 0.22	<0.001
LVIDs (cm)	2.68 ± 0.46	2.75 ± 0.67	3.48 ± 0.71	2.82 ± 0.40	<0.001
LVIDd (cm)	4.09 ± 0.56	4.62 ± 0.66	5.33 ± 0.71	5.17 ± 0.67	<0.001
LAVs (mL)	45.44 ± 5.69	51.41 ± 5.77	54.09 ± 5.3	46.04 ± 5.22	<0.001
LAVI (mL/m^2^)	29.89 ± 2.29	31.03 ± 2.03	29.72 ± 4.97	27.81 ± 2.27	<0.001
RVD1(cm)	3.54 ± 0.19	3.70 ± 0.27	3.89 ± 0.22	3.41 ± 0.24	<0.001
RAD (cm)	2.52 ± 0.22	2.78 ± 0.25	3.07 ± 0.42	2.65 ± 0.28	<0.001
RAV (mL)	24.82 ± 4.77	27.60 ± 5.06	28.17 ± 5.41	27.00 ± 4.67	0.006

Changes in dimensions of the left and right ventricles and atria in systole and diastole in normal pregnancy

As seen in Table 4, the mean values of the dimensions of the LVPWd and LVPWs in the second and third trimesters increased compared to the values in the first trimester of pregnancy. The mean difference between LVPWd in the first and second trimesters was 0.01, which was not a statistically significant difference (t = 0.61, p = 0.548); the difference increased to 0.08 in the third trimester, a statistically significant difference in mean (t = 2.18, p = 0.034).

**Table 4:** Changes in the left and right ventricular and atrial dimensions in systole and diastole in normal pregnancy among the study participants LVPWd (cm): left ventricular posterior wall in diastole, LAD (cm): left atrial diameter, LVPWs: left ventricular posterior wall in systole, LVIDs: left ventricular internal diameter in systole, LVIDd: left ventricular internal diameter in diastole, LAVs: left atrial volume in systole, LAVI: left atrial volume index, RVD1 (cm): right ventricular basal diameter (basal diameter), RAD (cm): right atrial diameter, RAV (cm): right atrial volume, CI: confidence interval

Cardiac parameters	Second versus first trimester		p-value	Third versus first trimester		p-value
	Mean difference (95%CI)	Paired t-test		Mean difference (95%CI)	Paired t-test	
LVPWd (cm)	0.01 (-0.03, 0.06)	0.61	0.548	0.08 (0.01, 0.15)	2.18	0.034
LAD (cm)	0.23 (0.17, 0.40)	9.92	0.001	0.33 (0.16, 0.79)	16.05	0.001
LVPWs (cm)	0.02 (-0.14, 0.09)	0.41	0.687	0.06 (-0.20, 0.08)	0.88	0.384
LVIDs (cm)	0.06 (-0.34, 0.22)	0.44	0.663	0.77 (0.58, 0.96)	8.08	0.001
LVIDd (cm)	0.52 (0.39, 0.64)	8.41	0.001	1.23 (1.08, 1.38)	16.65	0.001
LAVs (mL)	6.00 (4.24, 7.76)	6.86	0.001	8.20 (6.20, 10.21)	8.27	0.001
LAVI (mL/m^2^)	-1.05 (-1.79, -0.031)	-2.86	0.007	-0.46 (-1.19, 2.12)	0.57	0.575
RVD1 (cm)	0.17 (0.11, 0.24)	5.40	0.001	0.36 (0.28, 0.43)	9.44	0.001
RAD (cm)	0.27 (0.21, 0.33)	9.23	0.001	0.25 (0.23, 0.27)	5.70	0.001
RAV (mL)	3.14 (0.73, 5.55)	2.68	0.013	3.72 (2.34, 9.92)	2.73	0.001

In the postpartum period, the mean LVPWd was slightly higher (mean difference = 0.01) than the first trimester mean value; however, this difference was not significant statistically (t = 0.49, p = 0.626). The changes between the first and third trimester readings in most cardiac sizes were statistically significant (p < 0.05), except for LVPWs and LAVI, where the mean differences of 0.06 and -0.46, respectively, were not significantly different from the first trimester readings. Also, most cardiac sizes returned to values in the postpartum period, which were not significantly different (p > 0.05) from the first trimester values (Table 5).

**Table 5:** Changes in the left and right ventricular and atrial dimensions in systole and diastole in normal pregnancy among the study participants *Statistically significant LVPWd (cm): left ventricular posterior wall in diastole, LAD (cm): left atrial diameter, LVPWs: left ventricular posterior wall in systole, LVIDs: left ventricular internal diameter in systole, LVIDd: left ventricular internal diameter in diastole, LAVs: left atrial volume in systole, LAVI: left atrial volume index, RVD1 (cm): right ventricular basal diameter (basal diameter), RAD (cm): right atrial diameter, RAV (cm): right atrial volume, CI: confidence interval

Cardiac parameters	Second versus third trimester		p-value	Postpartum versus first trimester		p-value
	Mean difference (95%CI)	Paired t-test		Mean difference (95%CI)	Paired t-test	
LVPWd (cm)	0.06 (-0.01, 0.13)	1.72	0.092	0.01 (-0.04,0.07)	0.49	0.626
LAD (cm)	0.10 (-0.10, 0.80)	0.24	0.814	-0.38 (-0.31, -0.43)	-12.96	0.001*
LVPWs (cm)	0.03 (-0.13, 0.07)	-0.58	0.569	-0.08 (-0.19, 0.03)	-1.49	0.145
LVIDs (cm)	-0.73 (-0.47, -0.99)	-5.59	0.001*	0.10 (-0.12, 0.32)	0.94	0.357
LVIDd (cm)	0.72 (0.63, 0.80)	16.69	0.001*	1.06 (0.98, 3.09)	1.05	0.301
LAVs (mL)	2.51 (0.57, 4.44)	2.62	0.013	0.96 (-1.36, 3.28)	0.84	0.408
LAVI (mL/m^2^)	-1.60 (-3.11, -0.08)	-2.13	0.039	-2.35 (-3.22, -1.48)	-5.48	0.001*
RVD1 (cm)	0.18 (0.08, 0.27)	-3.79	0.001*	0.13 (-0.22, -0.004)	-2.99	0.005
RAD (cm)	0.29 (0.17, 0.42)	4.89	0.001*	0.15 (0.04, 0.26)	2.77	0.011
RAV (mL)	0.56 (-1.33, 2.46)	0.61	0.546	2.48 (0.22, 4.75)	2.47	0.033

## Discussion

The index study is a longitudinal study involving 50 healthy pregnant women in the first trimester at the antenatal clinic and the cardiology unit of the Federal Medical Centre, Yenagoa, Bayelsa State. The pregnant women were followed up through the course of their pregnancy until six weeks postpartum.

In this study, the mean age of the participants was 31.4 years. This was similar to those obtained by other studies conducted in Osogbo in Osun State, a southwestern Nigerian state, by Adeyeye et al. [1] and Akinwusi et al. [20], where the mean age of the participants was 28.2 ± 5.9 years and 20-35 years, respectively, although this was a cross-sectional age-matched control study. A lower mean age was obtained from the mean age of study participants in the research by Dapper et al. [21], where the mean ages of first trimester and second trimester subjects were 26.73 ± 5.59 and 26.67 ± 5.68 years, respectively, and the mean age was 32.91 ± 6.10 for third trimester study participants. These mean ages are all within the reproductive age of women.

The left ventricular posterior wall (LVPW) in both systole and diastole increased across the trimesters of pregnancy. The mean difference between the left ventricular posterior wall in diastole (LVPWd) in the first trimester and third trimester was statistically significant (p = 0.01). The left ventricular posterior wall (LVPW) in diastole and systole increased progressively across the trimesters but dropped at postpartum to a value similar to the first trimester value with no statistical difference (p = 0.6260 and p = 0.145, respectively). This trend is similar to what was seen in other studies [1,22]. In the study by Adeyeye et al. [1], the left ventricular posterior wall (LVPW) in the pregnant cohort was significantly higher than in the non-pregnant control (p < 0.05), although there was no significant difference across trimesters.

In the research by Simmons et al. [23], there was a mean increase of 11% in left ventricular wall thickness in normal pregnancy, and this decreased significantly in the postpartum period. The implication of this trend of rising left ventricular posterior wall in diastole (LVPWd) that does not quite return to normal after the pregnancy can be an indicator that subjects may develop hypertension and left ventricular dysfunction later. Therefore, there is a need to be followed up more closely in subsequent pregnancies. The left atrial diameter (LAD) was found to progressively increase and peak in the third trimester (3.84 ± 0.32 cm); this is similar to the findings from other studies [1,24,25]. This increase in LAD is caused by the increase in preload, which occurs in pregnancy in concert with increased blood volume, and these changes occur early in pregnancy and are reversed at postpartum. This implies that this structural change in LAD that occurs in normal pregnancy would reverse at postpartum when the high output states associated with pregnancy no longer exist.

The left atrial volume index (LAVI) and right ventricular basal diameter (RVD1) also showed similar trends. The left ventricular internal diameter (LVID) in both systole and diastole increased across the trimesters, peaking in the third trimester but dropping postpartum to a level higher than the first trimester value. Simmons et al. [23] also reported that the left ventricular internal diameter in diastole (LVIDd) increased slightly during pregnancy, but the left ventricular internal diameter in systole (LVIDs) did not change. The right atrial diameter (RAD) and right atrial volume (RAV) followed a similar trend of increasing throughout trimesters with differences statistically significant and then returning to almost baseline postpartum. However, the differences were statistically significant. These findings of progressive increases in left and right atrial dimensions in systole and diastole and those of the left ventricular internal diameter (LVID) and right ventricular diameter (RVD) across the trimesters were similar to the findings of the study by Adeyeye et al. [1] and others [1,25,26]. This increase in cardiac chambers was due to structural remodeling as a compensatory mechanism for chronic volume overload in pregnancy.

In a study by Savu et al. [27] involving 51 healthy pregnant women for whom serial echocardiography was done at the different trimesters and postpartum and with results compared with the echocardiography of 10 nulliparous women, there was a progressive increase in left ventricular end-diastolic dimension (LVEDD), left ventricular end-systolic dimension (LVESD), and left ventricular wall thickness in keeping with eccentric hypertrophy. Chamber diameters also increased longitudinally and transversely. All these changes reversed at three to six months postpartum [27].

Adeyeye et al. [1], in Ile Ife, Nigeria, concluded that cardiac chamber dimensions and left ventricular wall thickness, although within normal ranges, were significantly higher in pregnant Nigerian women than controls (non-pregnant women). Studies by Sanghavi and Rutherford [28] reported a similar increase in left ventricular end-diastolic dimension (LVEDD), left ventricular mass (LVM), and cardiac output. Cardiac output was 15% higher in twin pregnancy than in singleton pregnancy with a significantly higher increase in left atrial diameter consistent with volume overload [29].

Keser [14] reported an increase in left ventricular mass (LVM), left ventricular end-diastolic dimension (LVEDD), and left ventricular end-systolic dimension (LVESD) by 52%, 12%, and 20%, respectively, as well as interventricular septum during systole and diastole. The study by Schannwell et al. [26] described left ventricular hypertrophy during pregnancy. However, eight weeks after delivery, these changes were found to have returned to normal values.

Limitations of the study

Most pregnant women easily get tired during their echocardiographic assessment; as such, the operator has to be as fast as possible to relieve them before they experience fatigue. Moreover, the study could not ascertain the true baseline findings of the patients before their conception as the participants were selected in their first trimester.

## Conclusions

There are major adaptations of the maternal cardiovascular system that progress throughout normal pregnancy as a result of increased preload associated with pregnancy. The cardiac chamber dimensions in systole and diastole in the course of normal pregnancy progressively increased across trimesters and reversed significantly at six weeks postpartum. Some of these reversals at postpartum were to levels not approximate to but comparable to the first trimester levels. Cardiovascular changes in pregnant women should be considered in the assessment and management during the antenatal period even in those without comorbidities at booking. The data from this study can be used as a reference and a comparator for the diagnosis of disease states such as early cardiomyopathy.
